# A T1 and ECV phantom for global T1 mapping quality assurance: The T_1_ mapping and ECV standardisation in CMR (T1MES) program

**DOI:** 10.1186/1532-429X-18-S1-W14

**Published:** 2016-01-27

**Authors:** Gaby Captur, Peter Gatehouse, Peter Kellman, Friso G Heslinga, Katy Keenan, Ruediger Bruehl, Marcel Prothmann, Martin J Graves, Amedeo Chiribiri, Bernd Ittermann, Wenjie Pang, Reza Nezafat, Michael Salerno, James C Moon

**Affiliations:** 1 UCL Institute of Cardiovascular Science, University College London, London, United Kingdom; 2 Barts Heart Centre. St Bartholomew's Hospital, London, United Kingdom; 3 Cardiac MRI Department, Royal Brompton Hospital, London, United Kingdom; 4 National Heart, Lung, and Blood Institute, National Institutes of Health, Bethesda, MD USA; 5 Biomagnetics group, School of Physics, University of Western Australia, Crawley, WA Australia; 6 NeuroImaging group, MIRA Institute for Biomedical Technology and Technical Medicine, University of Twente, Enschede, Netherlands; 7 National Institutes of Standards and Technology (NIST), Boulder, MA USA; 8 Physikalisch-Technische Bundesanstalt (PTB), Braunschweig and Berlin, Germany; 9 AG Kardiale-MRT, Charité Campus Buch, Berlin, Germany; 10 Cambridge University Hospitals NHS Foundation Trust, Cambridge, United Kingdom; 11 Department of Cardiovascular Imaging, King's College, London, United Kingdom; 12 Department of Medicine (Cardiovascular Division) Beth Israel Deaconess Medical Centre, Harvard Medical School, Boston, MA USA; 13 Resonance Health, 278 Stirling Highway, Claremont, WA Australia; 14 University of Virginia Health System, Charlottesville, VA USA

## Background

Myocardial T1 and extracellular volume (ECV) estimates have applications in a range of myocardial diseases. Factors responsible for systematic inaccuracies in T1 mapping are beginning to be known^1-4^ but little is known about its delivery at ‘health-care system' scale and there is no global quality assurance (QA) system. Agarose phantoms are common in MRI and nickel ions preferred for lower temperature sensitivity^5^. This program aims to

**1** Create a partnership to design 1.5/3T phantoms for any manufacturer/sequence reflecting myocardial/blood T1 pre/post-contrast

**2** Test and mass produce phantoms to regulatory standards

**3** Distribute globally

**4** Analyse serial scans to understand T1 mapping at scale

**5** Publish recipes

**6** Explore delivery of a ‘T1/ECV Standard' via local calibration

We report results of steps 1-3.

## Methods

**Figure 1:**
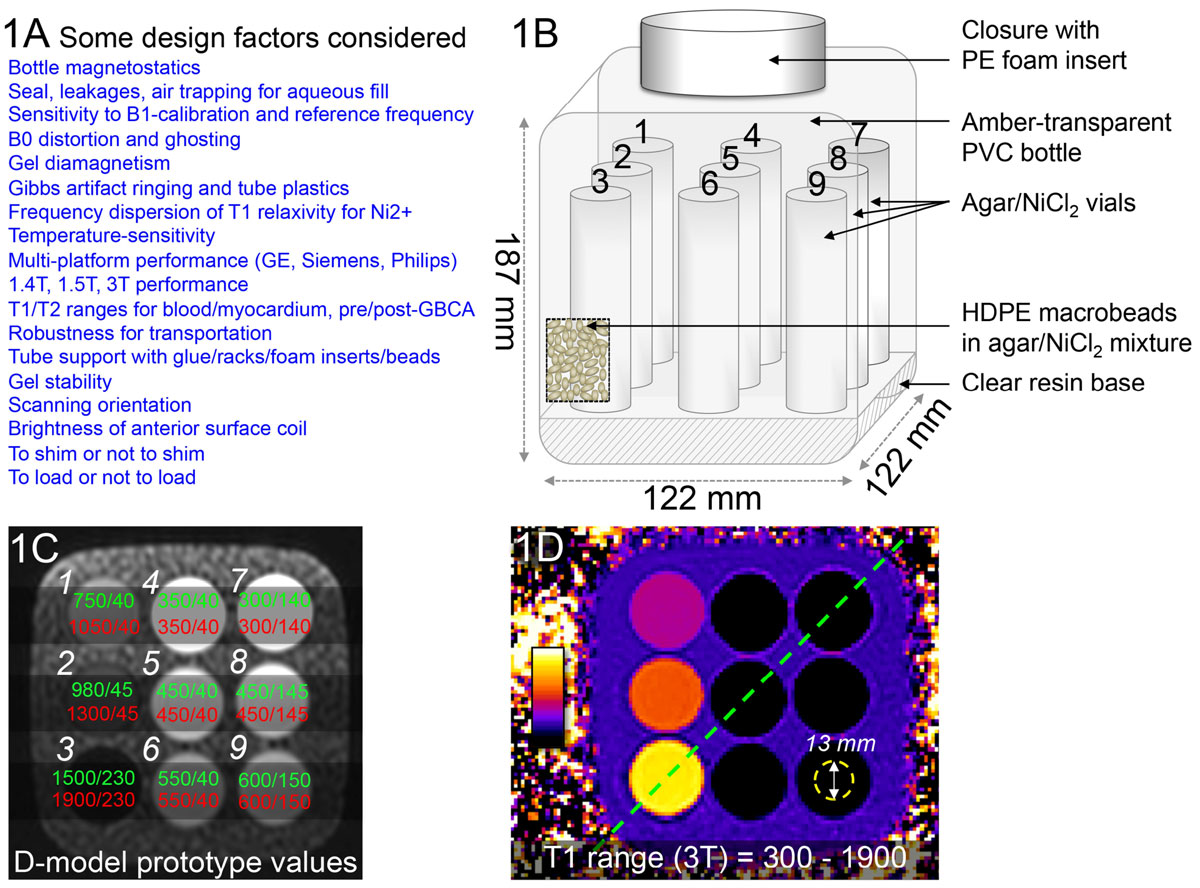
**1A Some of the critical design considerations**. **1B** Internal phantom structure showing tubes supported on a translucent unsaturated polyester and styrene resin base. **1C** Axial trueFISP localiser image of T1MES. T1 and T2 values in prototypes mimic those of myocardium and blood pre and post gadolinium based contrast agents at 1.5T in green and 3T in red. Relaxometry scopes are **1** Short native myocardium, **2** Long native myocardium, **3** Native blood, **4** Short postGBCA myocardium, **5** Medium postGBCA myocardium, **6** Long postGBCA myocardium, **7** Short postGBCA blood, **8** Medium postGBCA blood, **9** Long postGBCA blood. **1D** Typical T1 map of 3T prototype obtained by MOLLI using a bSSFP readout.

A design collaboration was created (clinicians/physicists/regulatory bodies/SME). After identifying critical design factors (Fig 1A) and discarding models with excessive *B*_*0*_*/B*_*1*_ distortion, the layout in Fig 1B was adopted. 9 tubes with differently doped agarose were embedded in a gel matrix and high-density polyethylene (HDPE) macrobeads added for *B*_*1*_ homogeneity. Tube diameter >20 mm was needed for regions of interest to exclude Gibbs artifacts. *B*_0_/*B*_1_ homogeneity was mapped to evaluate distortion. We hypothesised that dilution of dielectric permittivity by HDPE beads would reduce *B*_*1*_ inhomogeneity. This design was compared to ones using sodium chloride (*NaCl*) for increased conductivity, sucrose for reduced permittivity or poly methyl-methacrylate (PMMA) microbeads. Tubes with T1 = 250-1900 ms and T2 = 45-250 ms were reproducibly manufactured and separate ranges adopted for 1.5/3T (Fig 1C). 10 Prototypes were fabricated (5 each for 1.5/3T) for gold standard measurements: T1 by inversion-recovery spin echo(IRSE, 8 inversion times, 25>3200 ms); T2 by SE(8 echo times, 10>640 ms). Prototypes were then distributed to 9 experienced/regulatory centres for further testing.

## Results

**Figure 2:**
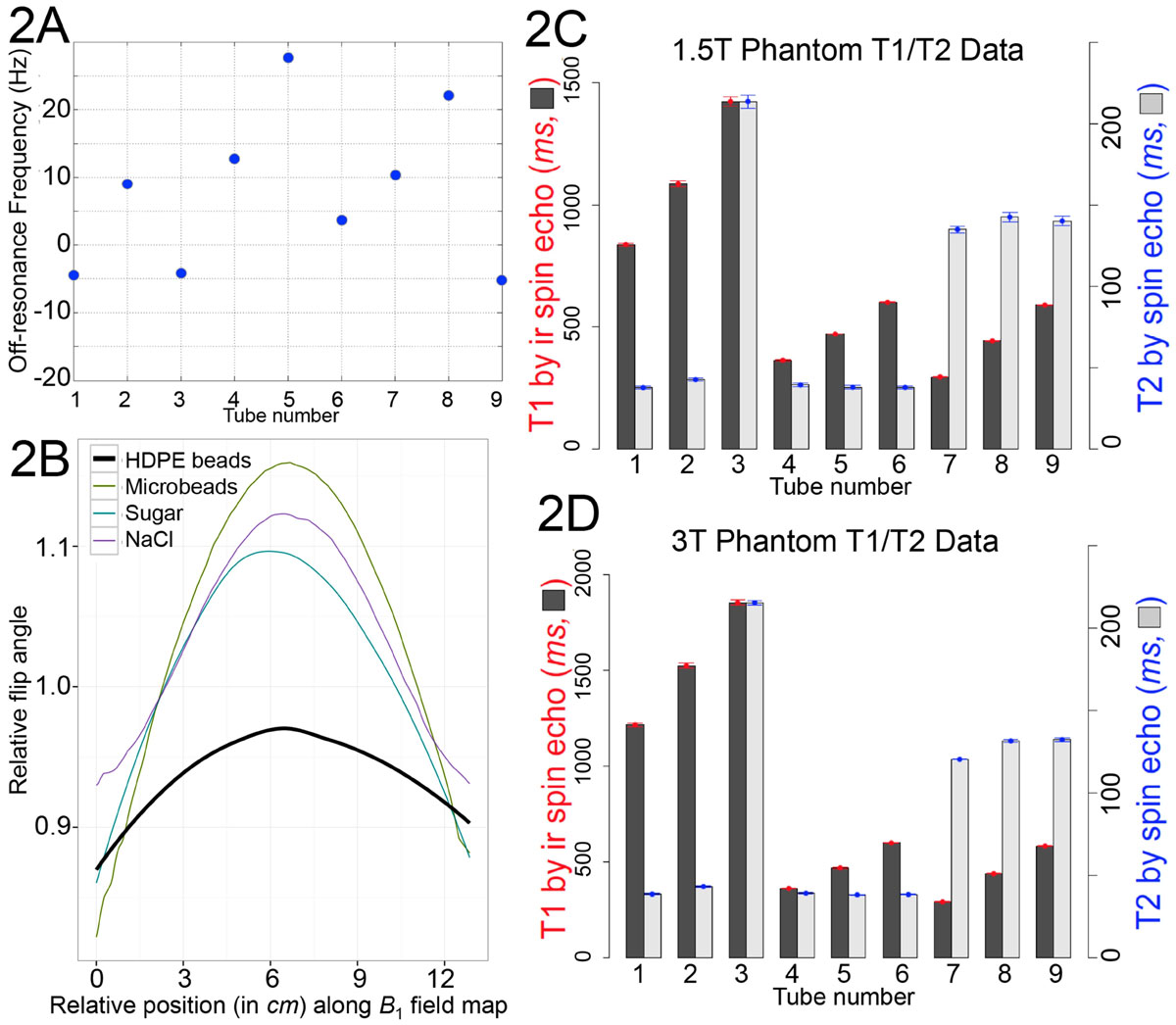
**2A** ***B***_***0***_ **field homogeneity across the 9 phantom compartments as a measure of off resonance in Hz at 3T**. **2B** Diagonal profile of the *B*_*1*_ field as per green line in **1D** comparing relative flip angles on Siemens 3T system. Variance of *B*_*1*_ was smallest across the 9 compartments with CoV 1.54% for HDPE beads measuring 2 to 4.8 mm. Highly monosized microbeads measured 6µm and were composed of crosslinked PMMA polymer. Neither microbeads, sucrose nor *NaCl* efficiently flattened the *B*_*1*_ field. **2C** and **2D** Variation in the mean T1 and T2 gold standard values and corresponding standard deviation shown as whiskers for all the D model prototype phantoms at 1.5 and 3T.

T1 maps were free from off-resonance artifacts (Fig 1D). The bottle geometry, coaxial with *z* and imaged transversely, showed < ± 0.3 ppm *B*_*0*_ uniformity (Fig 2A). HDPE beads flattened the *B*_*1*_ field at 3T (Fig 2B) especially compared to *NaCl*, sucrose and PMMA beads. T1 increased with temperature (0.19-1.54% change/°C) while T2 decreased(-0.93-1.45% change/°C). Comparison of gold standard values (Fig 2C,D) between prototypes confirmed reproducible manufacturing(coefficients of variation T1 0.97/1.35%, T2 1.25/2.73% for 1.5T/3T). Recipes were submitted for regulatory approval and manufacture will be complete by Sep'15.

## Conclusions

We created a collaboration to develop CE/FDA-approved phantoms for QA of T1 and ECV protocols. 70 revised phantoms with a multi-vendor user manual are now being distributed to centres worldwide for a 1-year academic exploration of T1 mapping sequences, platform performance and stability.

